# Cognitive Phenotypic Plasticity: Environmental Enrichment Affects Learning but Not Executive Functions in a Teleost Fish, *Poecilia reticulata*

**DOI:** 10.3390/biology11010064

**Published:** 2022-01-02

**Authors:** Giulia Montalbano, Cristiano Bertolucci, Tyrone Lucon-Xiccato

**Affiliations:** Department of Life Sciences and Biotechnology, University of Ferrara, 44121 Ferrara, Italy; lcntrn@unife.it

**Keywords:** behavioral plasticity, cognitive control, fish cognition, habitat complexity, individual differences

## Abstract

**Simple Summary:**

Environmental enrichment is extremely important for an individual’s neural, cognitive and behavioral development. Emerging animal models, such as teleost fish, may contribute to our understanding of enrichment-driven cognitive plasticity. We studied the cognitive consequences of living in enriched conditions in *Poecilia reticulata*. In particular, we compared subjects raised alone in a barren aquarium versus subjects exposed to enriched aquaria (with conspecifics, natural substrate, plants, and live prey) in three different cognitive tasks to measure learning, inhibitory control, and cognitive flexibility. Our results showed that guppies from the enriched aquaria learned a color discrimination faster compared to the subject raised in barren conditions. However, in the two remaining cognitive tasks, we found no effect from the treatment, suggesting that enrichment does not affect inhibitory control and cognitive flexibility. This study reveals that enrichment-driven plasticity affects only specific cognitive abilities.

**Abstract:**

Many aspects of animal cognition are plastically adjusted in response to the environment through individual experience. A remarkable example of this cognitive phenotypic plasticity is often observed when comparing individuals raised in a barren environment to individuals raised in an enriched environment. Evidence of enrichment-driven cognitive plasticity in teleost fish continues to grow, but it remains restricted to a few cognitive traits. The purpose of this study was to investigate how environmental enrichment affects multiple cognitive traits (learning, cognitive flexibility, and inhibitory control) in the guppy, *Poecilia reticulata*. To reach this goal, we exposed new-born guppies to different treatments: an enrichment environment with social companions, natural substrate, vegetation, and live prey or a barren environment with none of the above. After a month of treatment, we tested the subjects in a battery of three cognitive tasks. Guppies from the enriched environment learned a color discrimination faster compared to guppies from the environment with no enrichments. We observed no difference between guppies of the two treatments in the cognitive flexibility task, requiring selection of a previously unrewarded stimulus, nor in the inhibitory control task, requiring the inhibition of the attack response toward live prey. Overall, the results indicated that environmental enrichment had an influence on guppies’ learning ability, but not on the remaining cognitive functions investigated.

## 1. Introduction

Individuals of the same species might experience different environmental conditions if they live in different habitats or if the conditions vary with time [1]. When a single phenotype or evolved differences between populations are not adequate to cope effectively with spatiotemporal fluctuations in environmental conditions, we expect selection to favor the evolution of adaptive plasticity mechanisms. Plasticity may allow an individual to develop a phenotype that confers fitness advantages in specific environmental conditions [2]. For example, studies in *Daphnia* spp. have demonstrated that individuals develop a defensive helmet when they detect predator cues in their environment [3,4]. Subsequent studies have provided compelling evidence of phenotypic plasticity for a range of morphological [5,6,7], physiological [8,9,10], and behavioral traits [6,11,12].

Recently, interest in the plasticity of cognitive traits has been growing. Cognitive abilities can substantially affect animals’ fitness [13,14], and are involved in coping with changing environmental conditions [15,16]. Notably, cognitive abilities appear to be expensive to develop, as high metabolic requirements characterize the underlying neural tissues [17,18]. If an individual invests in the neural tissue necessary to achieve higher cognitive abilities, we expect the individual to compensate by reducing the investment in other tissues or functions. For example, guppies, *Poecilia reticulata*, artificially selected for increased brain size developed a shorter gut and demonstrated reduced investment in reproduction [18]. The trade-offs involving cognition make it possible to hypothesize the evolution of phenotypically plastic cognitive abilities that match the environment in which an individual develops and lives.

A well-studied example of cognitive plasticity is determined by a set of biotic and abiotic factors encompassing the presence of habitat complexities, social companions, and live prey, which are referenced collectively as environmental enrichment [19,20]. Environmental enrichment yields to brain changes and improved cognitive abilities in various species. In rodents, environmental enrichments, such as a large cage with social companions, toys, and hiding places, trigger brain plasticity, brain gene expression and, ultimately, cause an increase in learning and memory abilities [21]. In teleost fish, in which intense neurogenesis occurs throughout the life [22], research has been often linked environmental enrichment to cognitive abilities. For example, Salvanes and colleagues [20] found that juvenile *Salmo salar* reared in enriched environments, such as tanks with rocks and plants, display an increase in spatial learning abilities. Carbia and Brown [23] tested, with a spatial-learning task, individuals of Cocos frill-goby, *Bathygobius cocosensis* exposed to environments with or without rocks and found that fish from the enriched environment learned the task’s solution faster. Strand and colleagues [24] showed that performance of juvenile cod, *Gadus morhua*, in learning to choose between prey via observational learning is influenced by environmental enrichment, consisting of rocks and seaweed.

Considering that research has described a number of different cognitive abilities in fish [25,26,27], it is surprising that evidence of environmental enrichment plasticity is limited to learning tasks. In this work, we aimed to investigate environmental enrichment effects on a set of three cognitive abilities in a teleost fish, the guppy *P. reticulata*. Before the cognitive testing, we raised newborn guppies in either barren aquaria or aquaria enriched with social companions, gravel bottoms, natural and artificial plants, and live prey. The three cognitive abilities investigated were learning [28], cognitive flexibility [29,30,31], and inhibitory control [32,33]. We measured learning in a canonical color discrimination task. Cognitive flexibility and inhibitory control are part of a family of cognitive functions termed executive functions [34]. They have a large impact on animal life because they activate, in conjunction with other functions, to execute complex behavior and solve many cognitive tasks. Cognitive flexibility allows an individual to change a certain behavior or action for one more adapted to the situation [35]. We measured it with a reversal-learning task, in which the fish had to learn to choose the color that was not rewarded in the learning task. Inhibitory control describes an animal’s ability to inhibit a behavior or a response [34]. We used a procedure whereby the fish had to stop attacking a prey concealed behind a transparent barrier [32].

Based on prior studies, we expected that environmental enrichment would increase the subjects’ learning performance, e.g., [20,23,24]. The presence of this effect on learning would also serve as a control to ensure that the enrichment treatment triggered cognitive plasticity. The absence of prior studies on cognitive flexibility and inhibitory control does not permit us to formulate a strong prediction on the outcome of these tests. However, molecular studies have showed increased neural plasticity due to enrichment in fish [36], and in humans, research has reported an association between neural plasticity and inhibitory control [37]. Therefore, our main expectation would be to observe increased inhibitory control and cognitive flexibility in response to enrichment in guppies. 

## 2. Materials and Methods

### 2.1. Subjects

Overall, the study involved 39 juvenile guppies of a domestic strain (snake cobra green) collected on the day of their birth. We maintained the parents in standard 200-L aquaria equipped with biological filters and air pumps. Fluorescent lamps (30 W, 10,000 K) lit the aquaria with a 12 h:12 h light–dark cycle. We maintained the aquaria in a room with a controlled temperature (27 ± 1 °C) and fed the adult guppies twice per day with nauplii of *Artemia salina* and commercial flakes (Staple food Vipan, Sera, Heinsberg, Germany). The aquaria had a sex ratio of 50:50, and individuals could breed spontaneously. To minimize the risk of inbreeding, we routinely added 50 new guppies to the stock and relocated the guppies to different aquaria. 

### 2.2. Environmental Enrichment Treatment

**Figure 1:**
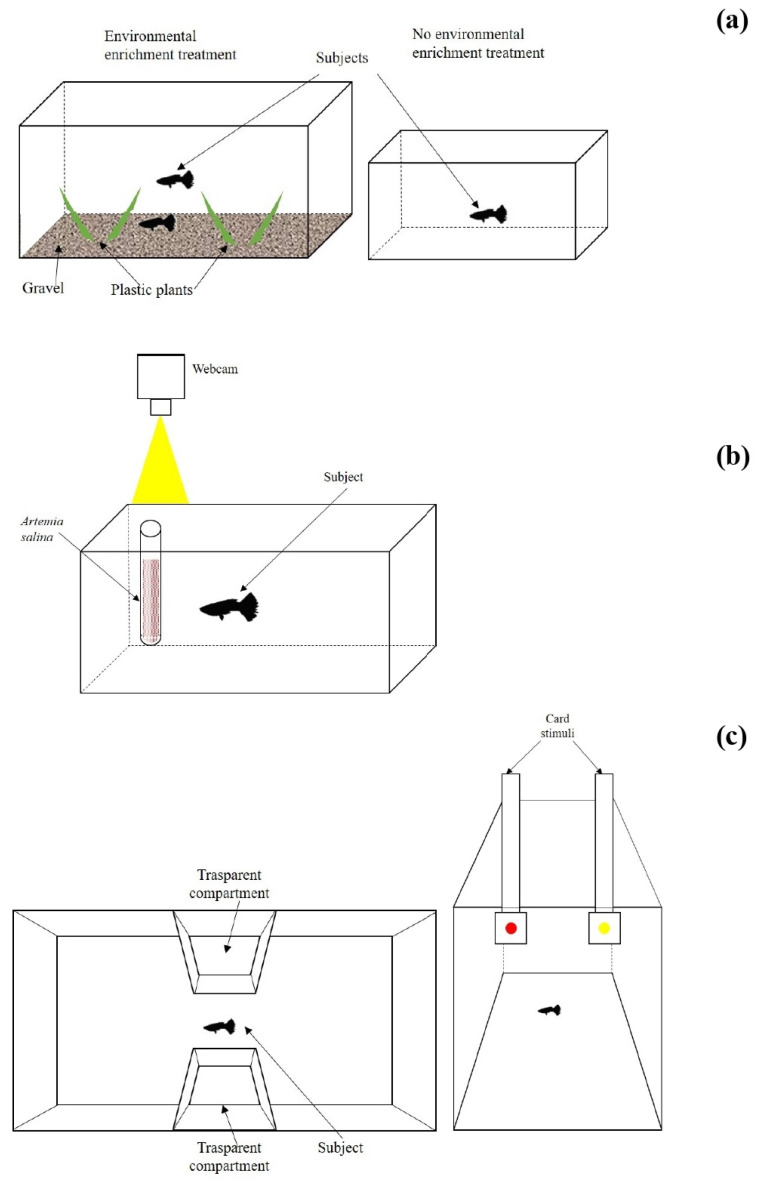
(**a**) Scheme of the aquaria used in the two treatments: the guppies of the enriched treatment (left) were housed in a 6-L aquarium with gravel bottom, plastic plants, and a conspecific, and they were fed with live *A. salina*; the guppies of the treatment with no enrichment were housed individually in a 2-L barren tank and fed with frozen *A. salina* (right). (**b**) Apparatus used in the inhibitory control task: the guppies were tested individually with a prey hidden inside a transparent tube. (**c**) Apparatus used in the learning task and in the cognitive flexibility task; the subjects were tested individually in the choice between two stimuli with different color, with a reward associated to the approach of the correct color.

We performed the environmental enrichment treatment on 39 subjects. To begin the treatments, we collected these guppies from the maintenance aquaria the day of their birth. We intended to start the subjects’ treatment as early as possible during the development, avoiding confounding effects due to uncontrolled experiences. As we kept the breeders in mixed-sex groups with free ability breed, we identified the newborn guppies by checking the maintenance aquaria daily. Upon collection, we randomly split the subjects into two groups for allocation to the two enrichment treatments (Figure 1a). 

We assigned a group of 18 subjects to the treatment with enrichment. We housed these subjects in 6-L plastic aquaria (32 cm × 16 cm, h = 14 cm; *n* = 18 aquaria). The aquaria’ walls were covered with green plastic. The first environmental enrichment consisted of the presence of a social companion (i.e., one newborn guppy of the same age). Beside the presence of a social companion, we provided further environmental enrichments. We fed the subjects with live prey (*A. salina*), administered twice per day. The subjects had to chase and capture the prey by swimming in the aquarium and peaking at the substrate, a situation that is relatively similar to foraging for live invertebrates in nature. Therefore, the administration of live prey consisted of a form of behavioral enrichment for the subjects [38,39,40]. In addition, we provided gravel and plastic plants to simulate a natural environment [19,20].

We assigned the remaining 21 subjects to the treatment with no enrichment. We housed these subjects individually in barren plastic aquaria (21 cm × 15 cm × h 11 cm; *n* = 21 aquaria). The aquaria’ walls were covered with green plastic. We fed these fish with dead *A. salina* twice per day. *A. salina* were killed with a freezing procedure, and were thawed before administration to the subjects. The frozen food ensured that the without-enrichment subjects had the same diet of the enriched-treatment subjects, but they were not exposed to the live prey.

For both experimental treatments, we changed water in the treatment aquaria every week. Each aquarium contained a small air stone to ensure water oxygenation. All remaining details of the treatment aquaria’ condition resembled those of the maintenance aquaria (temperature ~27 °C, photoperiod 12 h:12 h). The treatments lasted 30 days, a period that is sufficient to determine cognitive plasticity in this species [41].

### 2.3. Inhibitory Control

After 30 days of treatment [41], we subjected the guppies to the cognitive tasks. At this age, guppies are still juveniles as sexual maturation in this species occurs approximately at 3 months. We conducted the three cognitive tasks in the following sequential order: inhibitory control task, learning task, and cognitive flexibility task. This order reduced carryover effects across the tasks and exploited the contingency learned in the learning task as a starting point for the cognitive flexibility task (see below).

The inhibitory control task required the fish to inhibit the tendency to attack a live prey [32,33]. We conducted this task first, as it relied on a simple procedure and was less likely to cause carryover effects [42]. To perform the inhibitory control task, we initially housed each individual subject (*n* = 39) in a testing tank with green walls (4 L, 33 cm × 13 cm, h 15 cm; Figure 1b). Above the tank, we placed a transparent lid with a hole (⌀ 1.2 cm), through which we fed the fish, and on the day of testing, we presented the stimulus prey. We also installed a webcam 50 cm above the lid to record the trials. 

The procedure of this task consisted in two phases: habituation phase and test phase. The habituation phase lasted 3 days during which we fed the guppies with dry food mixed with water, increasing to 2-, 4-, and 6-times per day. The food was provided from the hole in the lid of the testing aquarium. We used dry food rather than live prey to ensure that the food items did not spread in the apparatus. In this way, the subject became habituated to receive the food from that specific area of the aquarium. In the last day of the habituation phase, we checked that all the subjects consumed the delivered food.

After the habituation phase, the subject began the test phase, in which we exposed it to a transparent tube (10 cm, ⌀ 1.2 cm) with a solution of 4 mL of live *A. salina*. We inserted the tube from the hole in the lid, and we blocked it to suspend vertically in the water column (Figure 1b). In an earlier study, we determined that the number of brine shrimp in the tube was 470 ± 48 (mean ± SD; *n* = 10; [42]). We expected the guppies to try to reach the prey, given the initial habituation to feed in correspondence to the hole in the lid. However, guppies could not reach the prey because of the transparent tube, and we expected this to cause the inhibition of predation behavior. To score inhibition, we recorded the behavior of each fish for 20 min and used the recordings to count the number of attacks toward the prey. We scored an attack when the fish touched the tube’s glass with its snout. Based on prior studies [32,33,42], we expected the fish to reduce the number of attacks over the testing time due to inhibition. Therefore, even if the test was continuous, we scored the data in 20-time bins of 1 min each. This allowed us to analyze the change in the number of attacks over the testing time. Fish with lower inhibitory control were expected to show a higher number of attacks and/or a smaller reduction in the number of attacks over time. 

### 2.4. Learning 

After the inhibition task, we randomly selected a subsample of 24 subjects (12 per treatment) to conduct the remaining tasks. This selection was necessary due to the logistic constraints associated with extensive training procedures. In the learning task, we trained the subjects to select a predetermined stimulus between two options, based on their color. We moved each subject to a larger glass tank (25 cm × 40 cm, h 25 cm) designed based on prior cognitive experiments in this species [43,44]. In this apparatus, we performed the learning and, thereafter, the reversal learning task (Figure 1c). This apparatus had an hourglass shape, with two main compartments, in which we presented the stimuli, and a central, narrow corridor. The corridor’s walls were transparent plastic, to allow the fish to see the entire apparatus and the stimuli from each position. The external walls of the apparatus were covered with green plastic to simulate vegetation and avoid interference from external stimuli.

For the color discrimination learning task, we initially administered a 2-day habituation procedure to the subjects. During the first day of habituation, we presented to the subjects with a transparent card (4 cm × 4 cm) by means of a transparent support on the aquarium’s wall. We inserted the card along one of the apparatus’s short walls (Figure 1c). To avoid startling the subject and to give it the time to approach the card spontaneously, we waited to insert the card until the subject was in the opposite side of the apparatus. On the card, there was a drawn yellow or red circle (⌀ 1.8 cm), which served as the stimulus. The color of the circle assigned to each subject corresponded to the reinforced color used in the following phases of the learning experiment. We predetermined the assignments pseudo-randomly and counterbalanced them between subjects from the different treatments; we tested half of the guppies with the red stimulus and the other half with the yellow stimulus. A statistical test run after completing the learning experiment indicated no effect of color on learning performance (χ^2^_1_ = 0.501, *p* = 0.480). After presenting the card stimulus, we waited until the guppy approached it. Upon approach, we released a mix of crumbled dry food water using a Pasteur pipette. As the scope of the habituation phase was to train the fish to approach the card, we delivered small quantities of food to attract it even when the fish was at a distance. We repeated the procedure of inserting the card and delivering the food eight times, with an interval of 15 min between each trial. We alternated the card’s presentation between the two short sides of the tank, and we varied the right-left position of the card. Only for this phase of habituation, we also housed 4 smaller guppies, randomly collected from the maintenance tanks, in the experimental apparatus [12,45]. This favored learning of the feeding schedule in the subject because, with the higher number of fish in the apparatus, it was more likely that one of them would notice the food and approach the card. Generally, the other fish rapidly followed and consumed the food. We removed these smaller guppies after this phase to avoid interference during the experiment.

On Day 2 of habituation, we subjected the guppies to 12 trials, in which we simultaneously introduced two stimuli with different colors, a card with a yellow circle and a card with a red circle. The two stimuli were placed as separate as possible on the tank’s short wall (Figure 1c). The fish had to choose and approach the card with the color assigned in the prior phase (i.e., rewarded color). As in the previous phase, we inserted the cards when the subject was in the opposite half of the tank. Therefore, the subject had to swim through the corridor and position itself in the center of the apparatus when choosing between the two cards. To obtain a robust measure of choice, from this phase onward, we considered the subject’s approach to the card when it reached a distance smaller than 0.5 body lengths. When the subject approached the card with the rewarded color, we considered it a correct answer, and we provided the food reward. In the case of a correct choice, after rewarding the subject, we removed the card with the incorrect stimulus, and we let the subject to consume the food before removing the card with the correct stimulus and terminating the trials. If the subject chose the card with the incorrect color, we waited until the subject chose the right card, we removed the incorrect stimulus, and we provided the reward. This might require multiple choices from the subject, but only until the maximum trial duration (15 min). After the subject consumed the reward, we terminated the trial as described above. In case of no choice (i.e., the fish did not approach any card), the maximum waiting time for a trial was 15 min. Then, we interrupted the trial and repeated it after an interval of 30 min.

After Day 2 of habituation, guppies started the test phase. We used only the data of this test phase to evaluate learning performance. The test phase consisted of a series of trials in which the fish could choose between the two stimuli. As described above, in each trial, we inserted both cards simultaneously in the aquaria during each trial, and we noted the choice of the fish. We administered food only when the subject chose the correct color (i.e., correct response) at first. If the subject chose the incorrect color, we removed both cards without delivering food. We administered 12 trials per day, but the number of test days varied according to each subject’s performance. We completed the testing when the subject reached a predetermined learning criterion of not more than seven errors in two consecutive days (<30% errors).

### 2.5. Cognitive Flexibility

We assessed cognitive flexibility with a reversal learning task. We presented the fish with the same color stimuli as in the prior task. However, to receive the food reward, the subject had to choose the card that was not associated with the reward during the prior learning task. Therefore, this task required the subjects to switch their learned preference between the two colors. The reversal learning task was administered to the 24 fish that completed the learning task, starting from day after each subject reached the learning criterion. One subject was discarded from the experiment because of an experimental error (final sample size = 23 subjects).

The reversal learning task took place immediately after each subject reached the criterion for the color discrimination learning task. We followed all the details of the procedure described for the test phase of the learning task. We administered 12 trials per day, until the subjects reached the criterion of maximum seven errors in two consecutive days (<30% errors). All other details of the procedure followed what was explained above. 

### 2.6. Statistical Analysis

For the statistical analysis we used R Statistical software (available at: https://www.r-project.org/ (accessed on 21 December 2021). All of the data showed normal distributions. As we dealt with a small number of independent variables (maximum 2) and they were important for the scope of the study, we calculated significance of the terms from full models (i.e., we did not conduct model selection). 

Initially, we analyzed the data of the inhibitory control task, which was composed by the number of attacks of each subject in each minute of the test. We applied Linear Mixed-Effects Models (LME; *lme* R function) to deal with the repeated measures. Beside the minute of testing, we fitted the treatment as fixed effect in the analysis; we also included subject ID as random effect to deal with the repeated measures. This analysis was expected to reveal differences in the overall number of attacks between the two treatments (main effect of treatment), as well as different trends in the reduction of attacks over time (interaction between minute of testing and treatment).

For the analysis of the learning task and the cognitive flexibility task, we adopted a similar approach. We analyzed the number of errors committed by each fish divided per each day of testing of the two learning experiments. As this latter data also included repeated measures, we used LME models fitted with the *lmer* R function to deal with differences in the number of training days between individuals. In these LMEs, we fitted the day of training (e.g., 1, 2, 3, …) and the treatment (enrichment versus no enrichment) as fixed effects; the subject ID was fitted as random effect. This analysis allowed us to detect differences in the overall number of errors to reach the learning criterion (main effect of treatment), as well as different learning trends across the two treatments (interaction between day of training and treatment).

## 3. Results

### 3.1. Inhibitory Control

Thirty-seven out of 39 guppies responded to the prey and attempted to capture them. The remaining two guppies, which were of the no-enrichment treatment, did not show interest for the stimulus prey and were removed from the dataset before the statistical analysis. Considering the 37 subjects that attempted to capture the prey, the overall number of attacks observed in the experiment was 166.24 ± 17.03 (mean ± standard deviation). The repeated measures LME indicated that the guppies decreased the number of attacks over testing time (minute of testing: F_1,735_ = 38.040; *p* < 0.001). Indeed, the number of attacks decreased from 29.46 ± 2.43 in the first minute of the experiment to 5.54 ± 1.06 in the last minute of the experiment.

**Figure 2:**
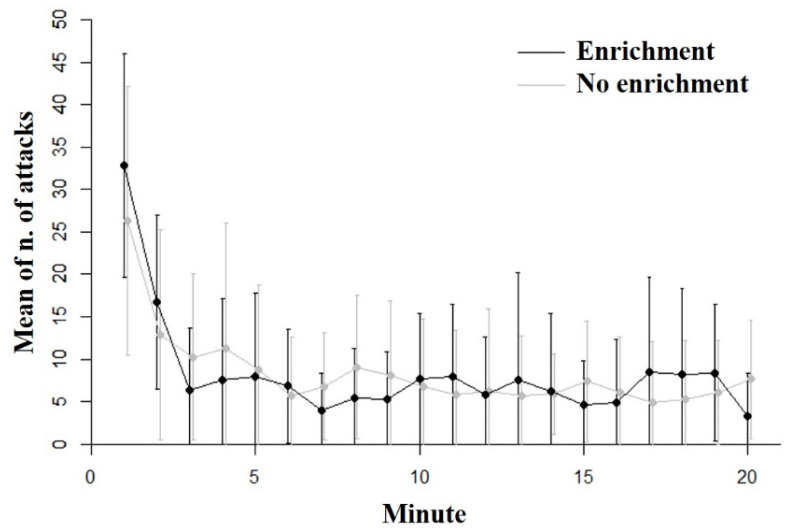
Results of the inhibitory control task. Number of attacks (mean ± SE) performed by the subjects of the two treatments in each minute of the test phase. The black line represents the treatment with environmental enrichment and the grey line the group without environmental enrichment.

The repeated measures LME did not detect significant differences in the number of attacks between the subjects of the enriched treatment and the subjects of the treatment without enrichments (F_1,735_ = 0.13; *p* = 0.722; Figure 2). In addition, there was no significant interaction between treatment and minute of the experiment (F_1,735_ = 0.78; *p* = 0.378).

### 3.2. Learning

**Figure 3:**
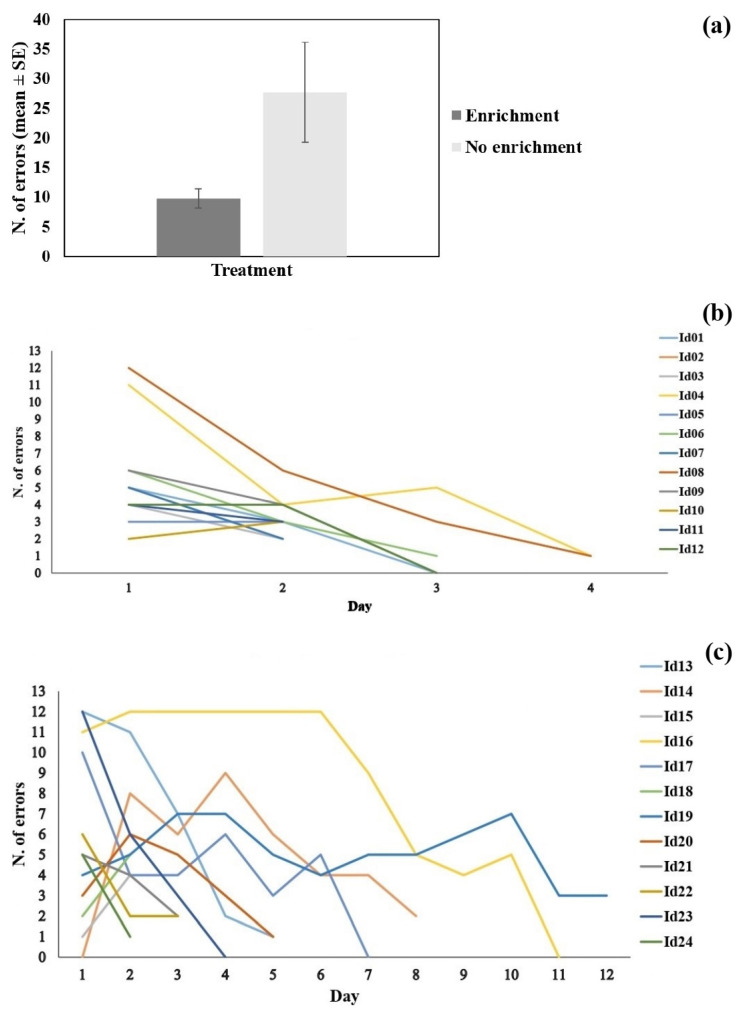
Result of discrimination learning task. (**a**) The number of errors to reach the learning criterion (mean ± SE) divided per treatment; the dark bar shows the subjects of the enriched treatment and the light bar the subjects without enrichment. (**b**) The number of errors made in each day of testing by the fish of the enriched treatment; each line corresponds to an experimental subject. (**c**) The number of errors made in each day of testing by the fish of the treatment without enrichment; each line corresponds to an experimental subject.

In the learning experiment, all the guppies acquired the color discrimination and achieved the learning criterion. Considering all of the 24 subjects tested in the color discrimination learning task, the number of errors necessary to reach the criterion was 18.92 ± 4.30, corresponding to 4 days of training (4.00 ± 0.57). The repeated measures LME analysis on the number of errors showed a significant difference between treatments (χ^2^_1_ = 5.118; *p* = 0.024; Figure 3a). The subjects of the treatment without environmental enrichment made more errors before reaching the learning criterion compared to the subjects of the treatment with environmental enrichment (Figure 3a). 

In the repeated measures LME analysis, the day of training also had a significant effect (χ^2^_1_ = 27.905, *p* < 0.001). More importantly, we found a significant interaction between treatment and day of training (χ^2^_1_ = 11.591, *p* < 0.001). This indicated that fish from the enriched treatment displayed a quicker decrease in the number of errors across training days (Figure 3b,c), and therefore they had greater learning performance.

### 3.3. Cognitive Flexibility

**Figure 4:**
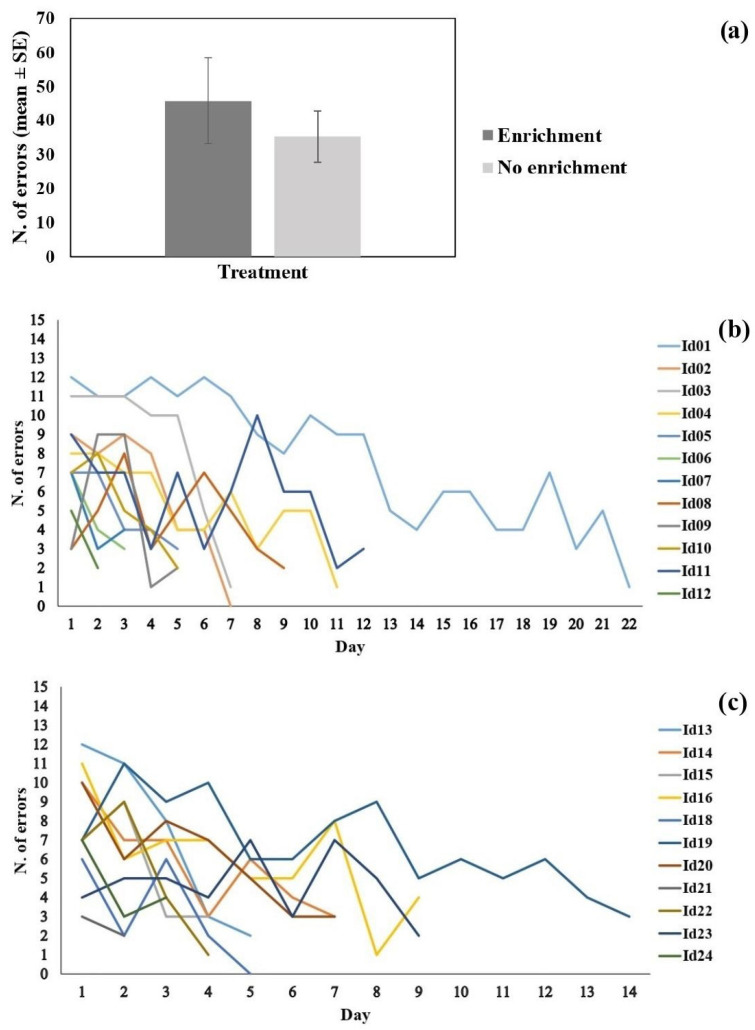
Result of the cognitive flexibility task. (**a**) The number of errors to reach the learning criterion (mean ± SE); the dark bar shows the subjects of the enriched treatment and the light bar the subjects without enrichment. (**b**) The number of errors committed in each day of testing by the fish from the enriched treatment; each line corresponds to an experimental subject. (**c**) The number of errors committed in each day of testing by the fish from the treatment without enrichment; each line corresponds to an experimental subject.

In the reversal learning task, all of the 23 guppies tested achieved the learning criterion, with an average number of errors of 40.69 ± 7.43, corresponding to 7 days of training (6.95 ± 0.95). The repeated measures LMM showed no significant difference between treatments in the number of errors committed before reaching the learning criterion (χ^2^_1_ = 0.401; *p* = 0.527; Figure 4a). The day of training had a significant effect on the number of errors (χ^2^_1_ = 100.637; *p* < 0.001; Figure 4b,c), indicating an overall decrease in the number of errors over testing days. Last, we did not find a significant interaction of treatment and day of training (χ^2^_1_ = 0.591; *p* = 0.442; Figure 4b,c).

## 4. Discussion

A relatively extended literature has demonstrated that animals plastically alter their cognitive abilities in response to the level of environmental enrichment they experienced (e.g., [21,41]). In teleost fish, research has mostly reported this plasticity for learning capabilities (e.g., [23]); in this study, we investigated whether other cognitive abilities might be similarly plastic. Using the guppy as a study species, we assessed the effects of environmental enrichment on cognitive phenotypic plasticity, focusing on three different traits: learning, cognitive flexibility, and inhibitory control. We found an effect due to the environmental enrichment on the learning performance, but not in the other cognitive abilities investigated (i.e., cognitive flexibility and inhibitory control). 

The effect of environmental enrichment on guppies’ learning plasticity aligns with the results of prior studies in a range of species, including teleosts such as the striped knifejaw, *Oplegnathus fasciatus* [46]; the Atlantic salmon, *S. salar* [20], the cod, *G. morhua* [24]; and the Cocos frill-goby, *B. cocosensis* [23]. These teleost species were assayed for various forms of learning using social and spatial tasks. Considering that our task involved learning in a different setting (i.e., when discriminating between colors), a possible interpretation for the whole literature on teleosts might be the presence of an effect of enrichment on a single learning function that is recruited when learning in many situations. Clearly, at the current stage of research it is important to obtain more knowledge on different learning tasks to confirm this interpretation.

Several studies have investigated the molecular mechanisms of the enrichment-driven learning plasticity [47,48,49], and one of these studies involved a fish species [20]. Evidence indicated that Atlantic salmon, *S. salar*, with increased learning due to enrichment also displayed upregulation in the expression of the transcription factor NeuroD1 [20]. NeuroD1 is widely conserved in vertebrates, e.g., [50] and plays a critical role in nervous system development [50] and plasticity [51,52,53]. Therefore, future research should investigate NeuroD1 as a determinant of learning plasticity due to enrichment in guppies, as well as in other species. Among the alternative pathways, those involving hormones should not be ignored. Many hormones have widespread effects on learning, e.g., [53], and it is known that enrichment may alter hormonal production in fish, particularly in relation to stress hormones [54,55,56]. Indeed, without enrichment, guppies might suffer several forms of stress such as that due to absence of appropriate substrate [55]. It is worth considering that the mechanisms of the observed learning plasticity might involve neuroanatomical changes in guppies’ brain. A recent study on this species found that individuals exposed to a spatially complex environment developed larger relative brain size and larger relative optic tectum size [36]. We also know that guppies with larger brain size display greater learning performance in discrimination tasks [18,57,58]. Therefore, brain anatomy changes might also have occurred in our guppies, although we cannot confirm this because we did not euthanize our fish for brain analysis.

More uncertainty remains on the ultimate causes of the observed plasticity in guppies’ learning performance. At least two hypotheses deserve attention in future investigations. First, in nature, enhanced learning abilities might be advantageous when the environment is more complex. If this is true, then the effect on learning observed in the laboratory studies, such as the present one, is potentially due to an adaptive mechanism of plasticity. We can also speculate that, for fish exposed to a barren environment, low investment in a learning function, such as the color discrimination investigated in our study, would make available additional resources for the development of other traits [18]. As a second hypothesis, it is worth considering that the plasticity observed in the guppies of the present study might be due to a nonadaptive mechanism. For example, a complex environment might provide enhanced stimulation to the neural system, and this might cause a developmental improvement of learning [59]. In support of this idea, came the studies on fish bred in hatcheries without enrichments and released in nature for conservation purposes. The behavior of these fish often appears maladaptive compared to that of wild fish [60]. The same may apply if the effect observed on guppies’ learning was due to stress hormones, as previously mentioned. 

A question that remains unanswered due to our experimental design is whether one or some elements of enrichment, but not others, affected guppies’ learning. Indeed, we simultaneously exposed the guppies of the enriched treatment to several stimuli (e.g., conspecifics and live prey). We did this in an attempt to obtain a larger and more robust effect, reducing the chances of type II errors. However, a study on lizards reported that social enrichment did not cause learning alterations [61]. Furthermore, in mice, a study revealed different effects on cognition according to the type of enrichment experienced by the subjects [62]. Similar variation in the effects of enrichment elements may occur also in guppies, and deserve investigations based on experiments that manipulate the elements one by one.

Regarding subjects’ inhibitory control and cognitive flexibility, we did not find an effect of environmental enrichment. The number of attacks toward the unreachable prey and the number of errors in the reversal learning tasks were substantially equal in the fish of the two treatments. Because inhibitory control and cognitive flexibility are considered part of a family of functions with distinct characteristics (i.e., the executive functions [34]), one may argue that, for this reason, they have no plasticity. However, at least two published studies in guppies contrasted this hypothesis. These studies found that social environment affects inhibitory control and that predation risk affects cognitive flexibility [63,64]. Moreover, results in our recent study of another teleost fish, the medaka, found plasticity of executive functions [65]. Therefore, we can conclude that the two executive functions examined in our study are, at least, potentially plastic, which raises the question of why enrichment did not affect inhibitory control and cognitive flexibility in our study on guppies. Considering that we found a clear effect of our enrichment treatment on the learning ability of guppies, we can exclude methodological problems in our experiment (e.g., the treatment was not long enough, or not in a critical developmental period). Overall, the most likely explanation for the absence of plastic changes in guppies’ inhibitory control and cognitive flexibility is that these specific executive functions do not respond to the specific environmental enrichment treatment that we performed. 

At the current research stage, it is only possible to speculate about the evolutionary causes of the lack of plasticity observed for the two executive functions (i.e., inhibitory control and cognitive flexibility) in juvenile guppies. Arguably, cognitive flexibility and inhibitory control are not necessary to deal with the environmental factors that determine enrichment, at least in the guppies. This is expected to cause relaxed selection on the environment-driven plasticity of these traits. In other words, guppies’ executive functions might not have the capability to change in relation to environmental enrichment. Before accepting this hypothesis, future research should investigate the effect of different enrichments on these and other executive functions in guppies to validate our findings. In this light, a comparison of studies in mice [66] and rats [41] reveals that these two rodents develop different cognitive flexibility responses in relation to the levels of environmental enrichment experienced. The same may occur in guppies, requiring further investigation with different enrichment treatments. We should not generalize the fact that we did not find plasticity of executive functions in guppies. Indeed, some aspects of executive functions vary substantially across vertebrate species [67], suggesting that there might be large interspecific variation in how executive functions respond to environmental enrichment. Possibly, other fish species will respond to environmental enrichment with marked plastic changes in executive functions. 

## 5. Conclusions

Collectively, the findings of the present study indicate that enrichment does not equally affect all the cognitive functions of juvenile guppies, but rather causes plasticity on specific traits. Future research requires a greater effort to increase the number of species investigated and to assess subjects’ performance in multiple cognitive tasks. This approach will help us to understand why enrichment only affects some cognitive functions and whether different species show different forms of plasticity.

## Data Availability

Data are submitted as Supplementary Materials: Data S1.xlsx.

## Supplementary Materials

The following supporting information can be downloaded at: www.mdpi.com/article/10.3390/biology11010064/s1, Data S1: dataset of learning, cognitive flexibility, and inhibitory control experiments.
